# Spatial relationship between evoked delayed potentials and deceleration zones of an isochronal late activation map in a patient with sarcoid-related ventricular tachycardia

**DOI:** 10.1016/j.ipej.2024.10.007

**Published:** 2024-11-04

**Authors:** Tomomasa Takamiya, Takashi Miyamoto, Shinsuke Miyazaki, Tetsuo Sasano

**Affiliations:** a Department of Cardiology, Saitama Prefectural Cardiovascular and Respiratory Center, 1696 Itai, Kumagaya, Saitama, 360-0197, Japan; b Department of Cardiovascular Medicine, Institute of Science Tokyo, 1-5-45 Yushima, Bunkyo-ku, Tokyo, 113-8519, Japan

**Keywords:** Cardiac sarcoidosis, Catheter ablation, Mapping, Ventricular arrhythmias

A woman in her early 50s with a history of cardiac sarcoidosis and an implantable cardioverter-defibrillator was referred for catheter ablation due to repetitive anti-tachycardia pacing therapy for sustained ventricular tachycardia (VT). A double extra stimulus from the right ventricular (RV) apex (S1,400 ms; S2,220 ms; S3,220 ms) induced clinical VT upon isoproterenol infusion. A 12-lead ECG showed monomorphic VT at a rate of 170 bpm, with a right inferior axis, right bundle branch block-like morphology, and positive concordance (Fig. 1A). After induction of VT, the left ventricular (LV) endocardium was entirely mapped using the CARTO system (Biosense Webster, Diamond Bar, CA) with a DecaNav mapping catheter (2-8-2-mm interelectrode spacing; Biosense Webster). Electrograms with anatomical information were manually collected during sinus rhythm, applying systematic RV extra stimuli (RVE) to identify evoked delayed potentials (EDPs) in an area presumed to be a VT substrate based on the cardiac MRI image, which showed late gadolinium enhancement with an aneurysm in the mid-lateral LV wall and clinical VT morphology. RVE was delivered with a coupling interval of 50 ms above the ventricular refractory period of 240 ms, and EDPs were defined as near-field potentials with a conduction delay >10 ms or block in response to RVE [1]. Electrograms were recorded at 340 LV sites during sinus rhythm. RVE was performed at 85 LV sites, and EDPs were revealed at 4 sites, but not at the other 81 sites. EDPs were clearly recorded at the mid-anterolateral LV wall, superior to the aneurysm (Fig. 2A and B). In Fig. 2A, a small, sharp component is visible at the end of an electrogram with a peak-to-peak bipolar voltage of 1.03 mV during sinus rhythm. This component was hidden in the far-field potential during right ventricular (RV) pacing at 500 ms (S1), delaying and separating during an RV extra stimulus with a coupling interval of 290 ms (S2). In Fig. 2B, a low-amplitude high-frequency signal is visible at the beginning of the electrogram with a peak-to-peak bipolar voltage of 2.34 mV during S1. The sharp signal was delayed and split during S2. Subsequently, radiofrequency catheter ablation was performed to eliminate the EDPs, performing pace mapping. The closest pace map was obtained near the EDP sites (Fig. 1B). Radiofrequency energy was delivered using an irrigated-tip ablation catheter (Thermocool Smart Touch; Biosense Webster, 35–40 W, 60 s per application) with a total radiofrequency time of 17 min. In addition, a local activation time map during sinus rhythm was delineated using an automated Wavefront Annotation algorithm (Biosense Webster). At sites with multicomponent or fractionated local electrograms, the automated algorithm typically annotates far-field potentials. Therefore, we reviewed all annotated local electrograms, manually reannotated the reproducible latest sharp deflections as necessary, and constructed an isochronal late activation map (ILAM) to display LV activation during sinus rhythm over 8 isochrones [2]. In the ILAM, the EDP sites were located in a deceleration zone with a large extent of isochronal crowding (Fig. 2C). The VT did not recur after the procedure.

**Fig. 1:**
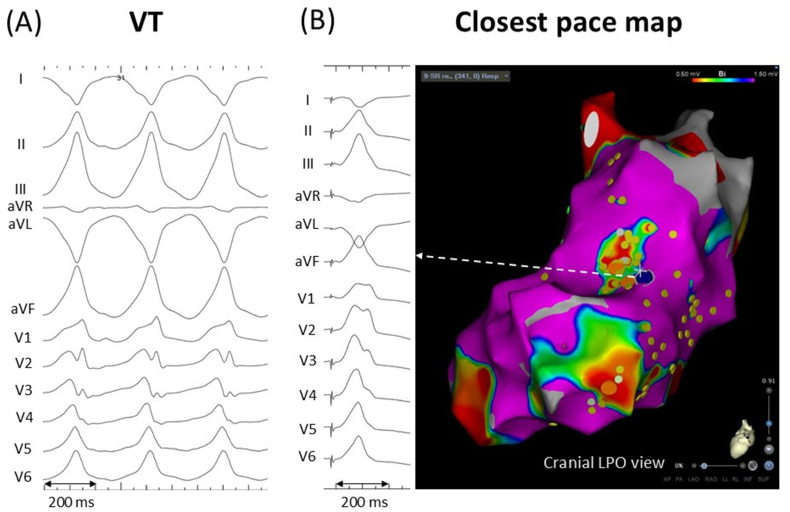
(A) ECG of clinical VT. (B) The closest pace map was obtained near the site where evoked delayed potentials were found. The blue tag shows the best pace map site.

**Fig. 2:**
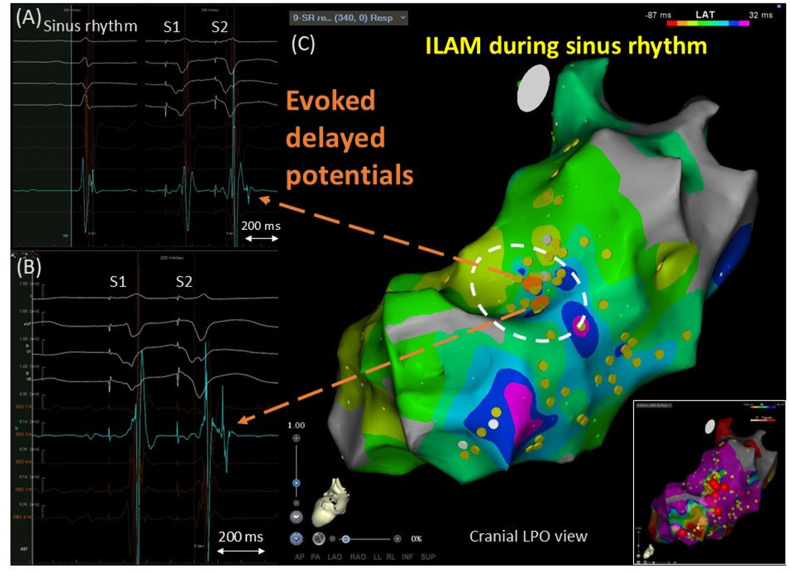
Evoked delayed potentials (EDPs) recorded in a deceleration zone of an isochronal late activation map (ILAM) during sinus rhythm. The EDPs (Orange tags) were located on the deceleration zone with a great extent of isochronal crowding in the left ventricular mid-anterolateral wall (surrounded by the white dotted line). Yellow tags denote other pacing sites with negative response to right ventricular extra stimuli. A bipolar voltage map with cutoff values of 0.5–1.5 mv and ablation tags (red) is attached.

Conducting a systematic annotation of EDPs in response to RVE aims to identify scar areas with functional conduction delays or blocks that are not detectable during sinus rhythm or continuous RV pacing [1]. Such electrograms with decremental conduction properties are associated with the diastolic pathway of post-MI VT [3], although little evidence is available regarding sarcoid-related VT. ILAM is a mapping strategy that displays conduction velocity in scarred areas. Deceleration zones identified during sinus rhythm can predict critical sites for scar-related VT [2]. These functional substrate mappings may improve the efficacy and outcomes of ablation. However, the spatial relationship between EDPs and deceleration zones of ILAM remains to be clarified. In this case, the obvious EDPs were exclusively identified on part of the deceleration zone in the left ventricular mid-anterolateral wall, and ablation targeting the EDP sites led to the abolition of the VT circuit. These findings support the possibility of complementary use of these approaches to efficiently determine successful ablation sites for VT.

## Ethical statement

Consent has been taken from the patient.

## Funding

This research did not receive any specific grant from funding agencies in the public, commercial, or not-for-profit sectors.

## Declaration of competing interest

The authors declare they have no financial interests.
